# Functionalized Graphene Sheets As Immobilization Matrix for Fenugreek *β*-Amylase: Enzyme Kinetics and Stability Studies

**DOI:** 10.1371/journal.pone.0113408

**Published:** 2014-11-20

**Authors:** Garima Srivastava, Kritika Singh, Mahe Talat, Onkar Nath Srivastava, Arvind M. Kayastha

**Affiliations:** 1 School of Biotechnology, Faculty of Science, Banaras Hindu University, Varanasi, India; 2 Nanoscience and Nanotechnology Unit, Department of Physics, Banaras Hindu University, Varanasi, India; RMIT University, Australia

## Abstract

*β*-Amylase finds application in food and pharmaceutical industries. Functionalized graphene sheets were customised as a matrix for covalent immobilization of Fenugreek *β*-amylase using glutaraldehyde as a cross-linker. The factors affecting the process were optimized using Response Surface Methodology based Box-Behnken design of experiment which resulted in 84% immobilization efficiency. Scanning and Transmission Electron Microscopy (SEM, TEM) and Fourier Tansform Infrared (FTIR) spectroscopy were employed for the purpose of characterization of attachment of enzyme on the graphene. The enzyme kinetic studies were carried out for obtaining best catalytic performance and enhanced reusability. Optimum temperature remained unchanged, whereas optimum pH showed shift towards acidic range for immobilized enzyme. Increase in thermal stability of immobilized enzyme and non-toxic nature of functionalized graphene can be exploited for production of maltose in food and pharmaceutical industries.

## Introduction

*β*-Amylase, distributed in higher plants and micro-organisms hydrolyze the *α*-1,4 linkages from the non-reducing ends of polyglucan molecules releasing *β*-maltose units. It is essential for the generation of maltose from cereal grain starch, thus making its role important in mashing and brewing process. The saccharifying activity is also exploited in bread making and in the use of malt as an additive in food stuffs. Maltose has properties such as mild sweetness, good thermal stability, low viscosity in solution and lack of colour formation, which accounts for its applicability in pharmaceutical dispensing and in the production of non-digestible sweetner (maltitol). Major use of *β*-amylase is in being the exclusive source of carbon in the production of diphtheria pertussis tetanus vaccine. The characteristic property of enzyme of yielding a single product maltose, is utilized in the structural studies of starch and glycogen [1], [2].

Enzymes being highly selective catalyze efficient reactions with few by products, thus they are environmentally favourable alternative to conventional chemical synthesis, particularly in the food and pharmaceutical industries where high reaction selectivity is essential. But, lack of long term operational stability, difficult recovery and reuse of the enzyme, limit their industrial applications. These limitations can often be overcome by immobilizing them on suitable matrices. The interaction between the enzyme and carrier material governs the properties of immobilized enzyme, thus, influencing the chemical, biochemical, mechanical and kinetic properties. The property of immobilized enzyme is also affected by particle mobility which is governed by particle size and solution viscosity [3].

Nano-materials and nano structures generally provide large surface area, low mass transfer resistance and high enzyme loading capability, which enables better interaction with the enzyme, resulting in increase in immobilization efficiency and enhancement in long term storage and recycling ability of the enzyme. Nanomaterials can also be engineered to present multiple surface functional groups for interacting with biomolecules. A wide range of nano materials have been studied and their positive effect on enzyme was shown through immobilization process [4]–[7].

Various graphene based nano materials have been used to fabricate functionalized biosystems integrated with nucleic acids, peptides, proteins and even cells. Graphene is a free standing 2D crystal with one atom thickness. This allotrope of carbon comprises layers of six atom rings in a honey combed network and can be conceptually viewed as a true planar atomic macromolecule. Graphene also gets benefitted by biological modification which improves its biocompatibility, solubility and selectivity. Proteins can exfoliate and modify graphene due to presence of various functional groups, through physical adsorption or chemical bonding [8]. Being, composed of carbon atoms, it does not alter the native biochemical properties of attached biomolecules significantly. Recently, material based on graphene have found use in technical applications such as biofuel cells, biosensing material, drug delivery and catalysis [9]–[12]. Issue of biocompatibility of graphene oxide has been studied on animal model and its non-toxic effect under low dose administration was demonstrated [13]–[16].

Response surface methodology (RSM) is a widely practiced approach for the production and optimization of different industrially important biotechnological and biochemical products. It is a collection of statistical and mathematical techniques useful for developing and optimizing processes. RSM is a method for constructing global approximations to system behaviour based on results calculated at various points in the design space. It also determines effect on specific response resulting from the changes in the level of the factors.

The choice of experimental design is affected by the shape of the experimental region, which is determined by the ranges of the variables. The Box-Behnken design is an independent rotatable or nearly rotatable quadratic design. It excludes the corners, where all variables are simultaneously at the maximum levels. The use of the Box-Behnken design is popular because it is an economical design and requires only three levels for each factor. Therefore, it requires few experimental combinations of the variables [4].

The basic objective of the present study include immobilization of Fenugreek *β*-amylase on functionalized graphene using RSM based Box-Behnken design of experiment and to study the effect of immobilization on kinetic parameters. Immobilized enzyme was characterized using SEM and TEM. The chemical interactions and bonding involved during immobilization were characterized by FTIR spectral analysis.

## Materials and Methods

Dry seeds of fenugreek (*Trigonella foenum-graecum*) were purchased from local market. The chemicals for buffers preparation were of Analytical or Electrophoresis grade from Merck Eurolab GmbH Damstadt, Germany. All other chemicals and reagents were purchased from Sigma Chem. Co. Milli Q (Millipore, Bedford, MA, USA) water with a resistance of higher than 18 MΩ cm was used all throughout the experiment.

*β*-Amylase was purified from dry seeds of fenugreek (*Trigonella foenum-graecum*) and had a specific activity of 732.59 units/mg. Homogeneity of the purified preparation was checked by SDS-PAGE [17].

### Protein assay

Protein concentration was determined by using Bradford's method (1976) [18], using crystalline bovine serum albumin as standard protein.

### Enzyme assays

#### Soluble enzyme assay

Enzyme activity was measured by following Bernfeld's method (1955) [19]. Reaction mixture was prepared by taking 0.5 mL suitably diluted enzyme and 1% starch prepared in 50 mM sodium acetate buffer, pH 5.0. This was incubated at 30°C for 3 min. Reaction was stopped by addition of 3,5 dinitrosalicylic acid. Test tubes were then placed in boiling water bath for 5 min and were allowed to cool down to room temperature, followed by addition of 10 mL of Milli Q water. Absorbance was recorded at 540 nm.

#### Immobilized enzyme assay

Graphene sheets cross-linked with Fenugreek *β*-amylase were incubated with 0.5 mL of starch solution (1%) prepared in 50 mM sodium acetate buffer, pH 4.5 for 3 min at 37°C, followed by centrifugation at 5000 rpm for 2 min at 4°C. Supernatant was pipetted out in test tube and 1 mL of 3,5 dinitrosalicylic acid was added, followed by placing it in boiling water bath for 5 min. It was diluted with 10 mL of Milli Q water before recording absorbance at 540 nm.

One unit of *β*-amylase is defined as the amount required for release of 1 µM of *β*-maltose per unit at 30°C and pH 5.0, under the specified condition.

### Immobilization efficiency

The immobilization efficiency was calculated using following formula:
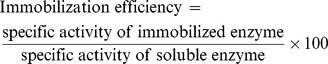
[1]


Enzyme units were calculated using starch as substrate, under standard assay conditions as described earlier. The immobilized protein was determined by subtracting the protein estimated in supernatant after immobilization from the total amount of protein used for immobilization.

### Functionalized graphene sheet preparation

Functionalized graphene sheets were prepared by thermal exfoliation of grahite oxide, following the protocol described by Staudenmaier [20], [21], [22]. Graphite powder (<50 µm, 1 g) was reacted with strong oxidising solution of conc. H_2_SO_4_, HNO_3_ and KClO_3_ at room temperature under constant stirring condition. The graphite oxide solution obtained was washed with distilled water and 10% HCl solution for removal of sulphate and other ionic impurities. It was then dried under vaccum at 80°C. The graphite oxide powder was placed in an alumina boat and inserted into a 1.5 m long quartz tube with outer diameter of 25 mm. The sample was flushed with Ar gas for 15 min followed by insertion of quartz tube into a tube furnace pre heated at 1050°C and was kept in it for 30 sec. The functionalized graphene sample was allowed to cool down to room temperature under Ar gas flow. Thermally exfoliated functionalized graphene thus prepared, was light weighted shiny black powder which differed in appearance from brownish graphite oxide. The sample prepared was suspended in double distilled water and was sonicated at room temperature for 10 min. It was then left undisturbed for 30 min for the settlement of the larger non exfoliated flakes and the suspended functionalized graphene was collected.

### Enzyme immobilization

The enzyme was coupled to the functionalized graphene with the help of glutaraldehyde as a cross linker. A preparation of 1 mg/mL of graphene was made by dissolving functionalized graphene in 50 mM sodium phosphate buffer (pH 7.0), which was divided into 17 aliquots according to the design of the experiment (Table 1). These aliquots were equilibrated in the buffer (1 mL reaction volume) overnight, followed by thorough rinsing with the same buffer. It was then treated with glutaraldehyde and kept in dark for 4 h at room temperature. After treatment it was washed with the buffer followed by incubation with the enzyme under dark condition for 12 h at 4°C. The immobilized enzyme was washed thoroughly with chilled buffer. Each step of immobilization was followed by thorough washing with chilled buffer and centrifugation at 5000 rpm for 2 min at 4°C. The response corresponding to the list of independent variables based on experimental design is shown in the Table 1. The immobilized enzyme was assayed by following procedure as described above.

**Table 1:** Box-Behnken experimental design for independent variables and their corresponding response (% immobilization).

Run	Glutaraldehyde	Functionalized	Enzyme	Immobilization	(%)
	(% v/v)	graphene (*µ*g)	(*µ*g)	actual	predicted
**1**	1.50	1500.00	600.00	59.20	58.98
**2**	3.00	1000.00	900.00	64.89	65.31
**3**	2.25	1000.00	600.00	79.92	81.63
**4**	2.25	1000.00	600.00	83.86	81.63
**5**	3.00	500.00	600.00	53.26	53.48
**6**	3.00	1000.00	300.00	41.08	41.73
**7**	1.50	500.00	600.00	54.90	56.18
**8**	2.25	500.00	900.00	66.62	65.99
**9**	2.25	1000.00	600.00	82.23	81.63
**10**	2.25	1500.00	300.00	61.27	61.90
**11**	1.50	1000.00	900.00	51.96	51.31
**12**	2.25	1000.00	600.00	81.00	81.63
**13**	2.25	1000.00	600.00	81.14	81.63
**14**	3.00	1500.00	600.00	83.10	81.82
**15**	1.50	1000.00	300.00	36.00	35.58
**16**	2.25	500.00	300.00	41.04	40.18
**17**	2.25	1500.00	900.00	74.54	75.40

### Characterization

The characterization studies of functionalized graphene sheets (both native and coupled) wereperformed by Transmission Electron Microscope (TEM, Technai 20 G, 200 kV), Scanning Electron Microscope (SEM, Philips: XL20) and Fourier Transform Infrared Spectroscopy (FTIR, Perkin Elmer Spectrum 100 instrument). TEM studies were done by placing a drop of sample on electron microscope 200 mesh copper grid and the water was allowed to get evaporated for complete dryness of the sample, followed by its loading into machine. SEM studies were carried out by sprinkling the samples on the stub having layer of silver glue for striking the particles. Secondary electron imaging mode was used for obtaining fine structural details. FTIR was performed in the range of 650 to 4000 cm^−1^ wave number. A good signal to noise ratio was achieved with 100 scans of each sample.

### Experimental set up and statistical analysis

The experiment was designed using Box-Behnken design for analysis and optimization of the factors (amount of functionalized graphene, concentration of glutaraldehyde and amount of enzyme) which influence the process. The initial values of the factors affecting the process of immobilization were determined by carrying out some preliminary experiments (data not shown). ‘Design Expert’ software (version 8.0, Stat-Ease Inc., Minneapolis, USA) was used for experimental design and analysis. The Box-Behnken design used for fitting the quadratic model led to the experiment consisting of 17 trials and all the factors studied were varied at three different levels. The variables and their levels selected for carrying out immobilization of Fenugreek *β*-amylase onto functionalized graphene were: amount of graphene (500, 1000 and 1500 µg); concentration of glutaraldehyde (1.5%, 2.25% and 3%) and amount of enzyme (100, 200 and 300 µg). The quadratic polynomial equation relating the variables to the responses was used for calculation of the mathematical relationship:


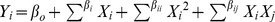
 [2]

Where, *Y_i_* is the predicted response, *X_i_X_j_* are input variables which influence the response variable *Y*; *β_o_* is the offset term; *β_i_* is the *i* ^th^ linear coefficient; *β_ii_* the *i* ^th^ quadratic coefficient and *β_ij_* is the *ij* ^th^ interaction coefficient. The co-efficients were estimated by performing the 17 trials and the generated mathematical model was validated by carrying out experiment at given optimal condition. The model was statistically analysed for evaluation of the anlaysis of variance (ANOVA), which determines the validity of the model based on Fischer's F-test, associated probability, correlation coefficient R and lack of fit test.

### Steady state kinetics

In all cases, control experiments using soluble enzyme were carried out.

The effect of pH on immobilized Fenugreek *β*-amylase was studied in the pH range of 3.0–9.0 (glycine-HCl: pH 3.0–4.0, acetate buffer: 4.0–5.5, sodium phosphate: 5.5–7.0, Tris-HCl: 7.2–9.0). 50 mM buffers were used in each case and assay procedure was performed under standard conditions. The optimum temperature was studied by carrying out assay procedures at temperatures between 25–75°C. The thermal stability was determined by incubating the enzyme at 55°C for different time intervals, followed by residual activity assay under standard condition. Starch concentration was varied in the range of 0.5–10 mg/mL for observing effect of substrate concentration on immobilized enzyme. Data thus obtained was used for calculation of *K_m_* and *V_max_* from Lineweaver-Burk plot. For the assessment of reusability, the immobilized enzyme was used for carrying out assay procedure repeatedly. After each assay, the immobilized enzyme was washed with 50 mM sodium phosphate buffer, pH 7.0. The aliquots of functionalized graphene coupled with enzyme were stored at 4°C and the residual activity was determined during 4 months storage.

## Result and Discussion

### Process optimization via RSM

For obtaining optimal conditions for enzyme immobilization, RSM was employed. Central Composite design and Box-Behnken design are the two designs of experiment commonly utilised in RSM. A comparative study between the two designs of experiment was done by Ferreira et al (2007) [23] and Box-Behnken design was found to be more efficient. In the present study, we have utilised the Box-Behnken design of experiment for optimization of response. Preliminary experiments were carried out for selecting operational ranges of variables having favourable effect on response. Experiment was designed within these ranges of parameters. The detailed results of experiment are presented in Table 1 as actual immobilization percentage (+0.5).

**Figure 1:**
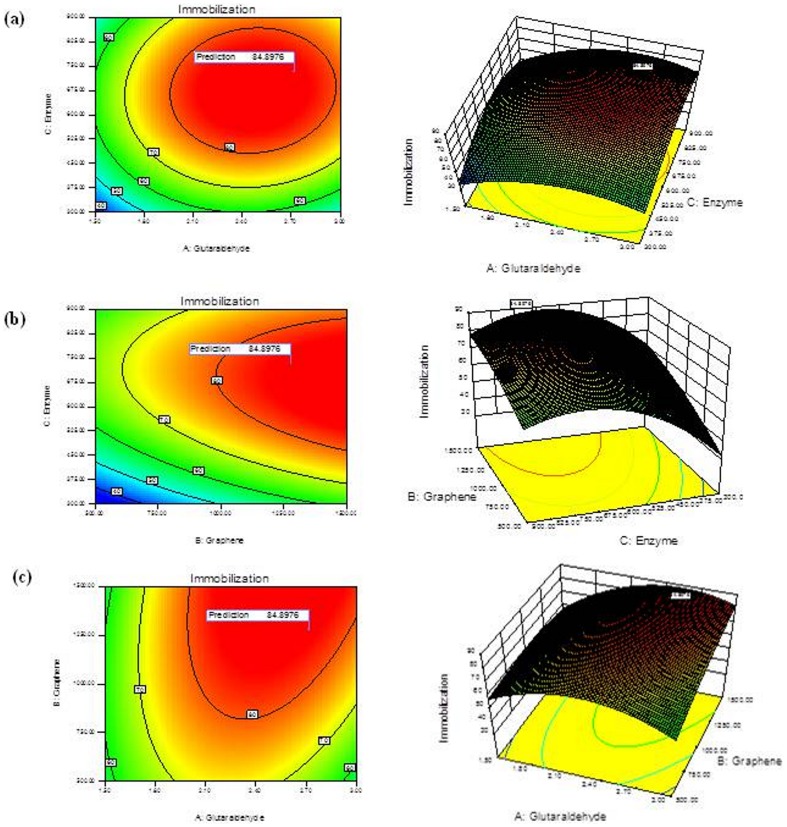
Response surface and contour plots showing effects of various parameters on immobilization (%) and the predicted optimal response. 3D and contour plot for (a) the effect of amount of enzyme and concentration of glutaraldehyde on immobilization (%), (b) the effect of amount of enzyme and amount of functionalized graphene and (c) the effect of amount of functionalized graphene and concentration of glutaraldehyde on immobilization (%).

These experimental results formed the base for building of model for obtaining maximum immobilization response as presented in Fig. 1 (Fig. 1(a) shows 3D and contour plot for the effect of amount of enzyme and concentration of glutaraldehyde, Fig. 1 (b) demonstrates the effect of amount of enzyme and amount of functionalized graphene and Fig. 1 (c) shows the effect of amount of functionalized graphene and concentration of glutaraldehyde, along with their interaction on immobilization efficiency). Following values of the factors were determined, functionalized graphene: 1277.78 µg, glutaraldehyde: 2.72%, enzyme: 733.33 µg, immobilization: 84.90%. For validation of the predicted values, experiments were carried out and 84.12% immobilization was achieved, which was in agreement with the predicted values. Multiple regression analysis was applied to establish the polynomial coeffeicients. A relationship was established by means of a quadratic polynomial equation. The final equation for determination of the immobilization efficiency can be summarised as follows:
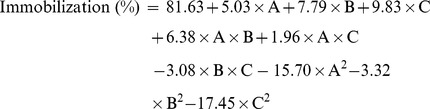
where A, glutaraladehyde concentration (% v/v); B, functionalized graphene (*µ*g) and C, enzyme (*µ*g).

For establishing the adequacy and significance of predicted quadratic model, ANOVA was performed (Table 2). The Model F-value of 209.34 implies the model is significant and there is only a 0.01% chance that this value could occur due to noise. The comparison of residual error (mean square) to the pure error (mean square) is done by lack of fit. The F-value of 1.03 implies the lack of fit is not significant relative to the pure error. The non-significant value of the lack of fit defines suitability of the model. The “Pred-R-Square” of 0.9710 is in agreement with “Adj-R-Square” of 0.9915. The “Adeq Precision” measures the signal to noise ratio and a value greater than 4 indicates adequacy. The designed model was found to be significant having the value of 40.199.

**Table 2:** Analysis of variance (ANOVA) for response surface model pertaining to percent immobilization.

Source	Sum of Squares	Df	Mean Square	F-Value	p-value	(Prob>F)
**Model**	4237.12	9	470.79	209.34	<0.0001	significant
**A-Glutaraldehyde**	202.71	1	202.71	90.14	<0.0001	
**B-Graphene**	485.01	1	485.01	215.66	<0.0001	
**C-Enzyme**	772.64	1	772.64	343.56	<0.0001	
**AB**	163.07	1	72.51	<0.0001		
**AC**	15.41	1	6.85	0.0345		
**BC**	37.88	1	16.85	0.0045		
**A^2^**	1037.85	1	461.48	<0.0001		
**B^2^**	46.27	1	20.57	0.0027		
**C^2^**	1281.75	1	569.93	<0.0001		
**Residual**	15.74	7	2.25			
**Lack of Fit**	6.85	3	2.28	1.03	0.4699	not significant
**Pure Error**	8.89	4	2.22			
**Cor total**	4252.86	16				

### Characterization

**Figure 2:**
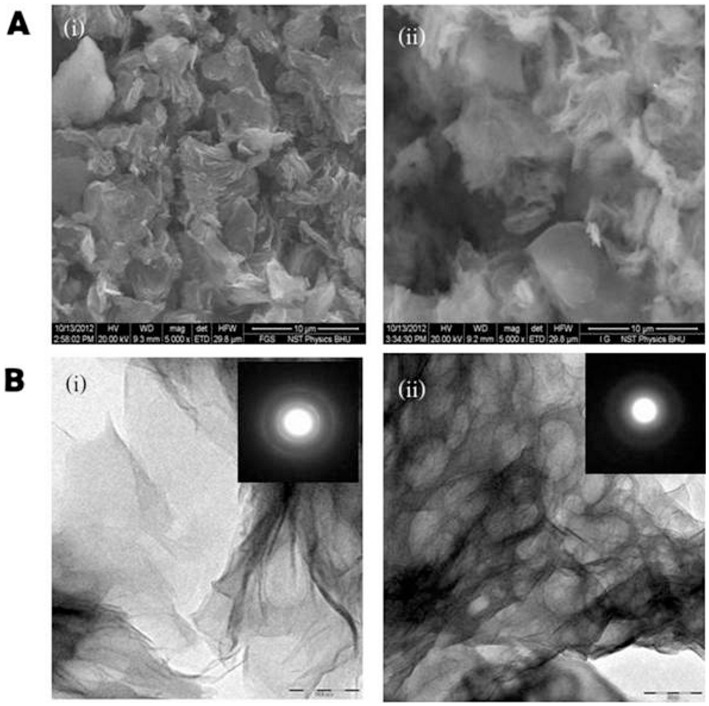
Micrographs of functionalized graphene sheets and the sheets having enzyme immobilized on them. (A) SEM images of (i) functionalized graphene sheets and (ii) enzyme immobilized on graphene sheets (B) TEM images of (i) functionalized grapheme sheets and (ii) immobilized enzyme with inset showing the selected area electron diffraction pattern.

**Figure 3:**
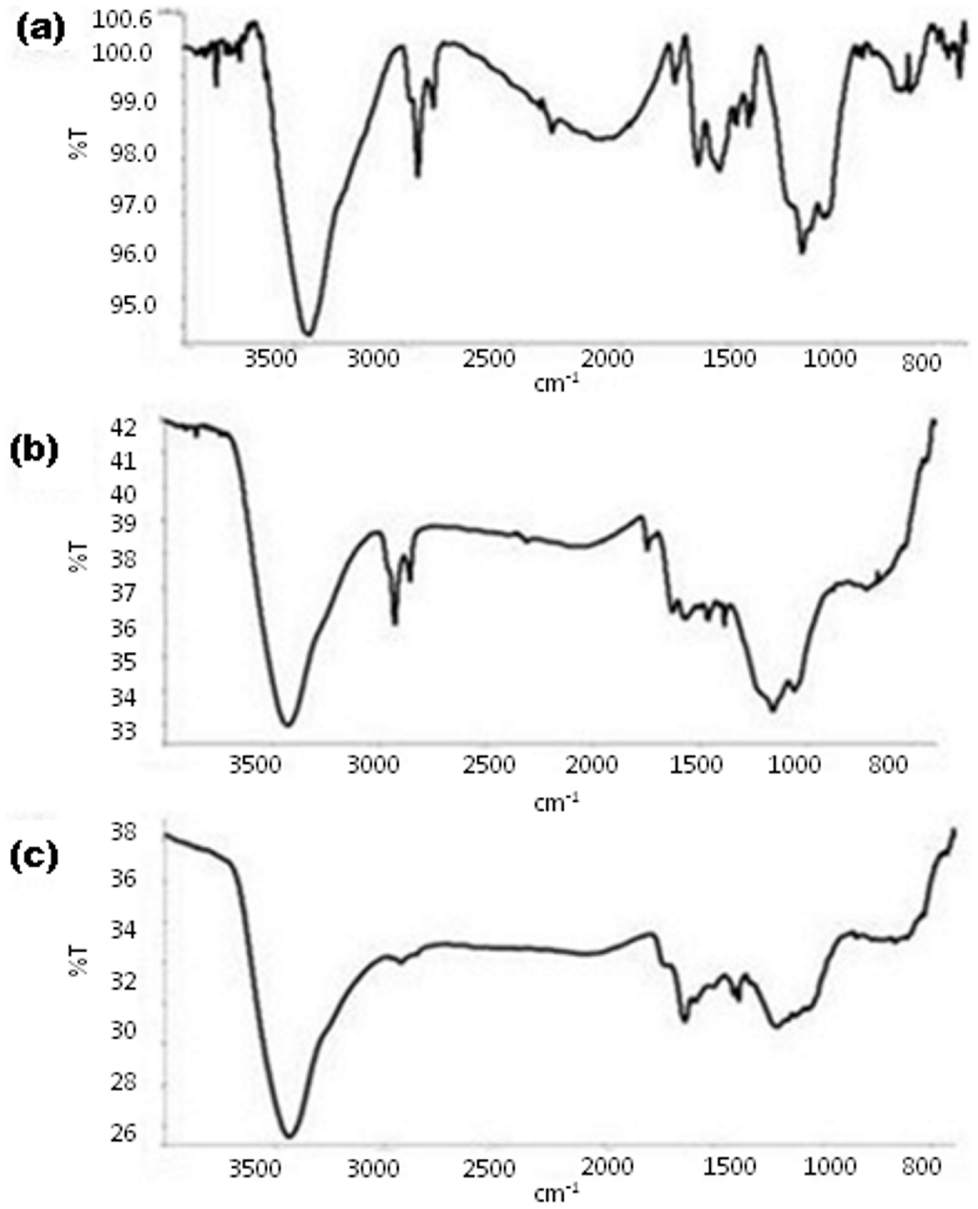
FTIR spectra of graphene sheets during different stages of immobilization. FTIR spectra of (a) enzyme immobilized on graphene, (b) glutaraldehyde treated graphene and (c) functionalized graphene sheets.

Systematical characterization of the enzyme immobilized on functionalized graphene was done by carrying out SEM and TEM, followed by FTIR spectroscopy. Bright field TEM images showed typical transparent sheets of functionalized graphene at 50 nm resolution, the characteristic Selected Area electron Diffraction (SAD) pattern obtained is shown in inset of Fig. 2. Immobilized enzyme can be seen in the TEM images of the functionalized graphene sheets after immobilization process. An alteration in characteristic SAD pattern of functionalized graphene was also observed due to immobilization of enzyme. SEM images also show distinctly the attachment of enzyme, presenting evidence in support of TEM images (Fig. 2). To get an insight of the interaction of the functionalized graphene with enzyme molecule, FTIR spectra were taken for native, glutaraldehyde treated and enzyme immobilized graphene sheets. The three FTIR spectra obtained showed difference in the vibration peaks corresponding to functionalized graphene and functional classes during different stages of immobilization (Fig. 3).

The natural graphite shows a characteristic peak located at 1625 cm^−1^ (C = C band), which has been assigned to skeletal vibrations of graphitic domains. Functional groups present in graphite oxide (e.g. hydroxyl, epoxide and carboxyl groups) are utilized in its further functionalization. The peak at 1740 cm^−1^ is characteristic of −COOH (−C = O in carboxylic acid) which is known to appear after execution of oxidation process. The O-H stretching mode of −COOH groups and −OH groups attached on the surface of functionalized graphene leads to appearance of peaks of O-H at ∼1400 cm^−1^ together with the band at 3450 cm^−1^. The vibration of C-O in carboxylic acid groups or epoxy groups results in appearance of the peak at ∼1054 cm^−1^. The cross linking of functionalized graphene has also been characterized by FTIR. Peaks corresponding to the C-H stretch modes of the cross linked molecule was located at 2927 and 2858 cm^−1^. An increase in the relative intensity between peaks at 1054 cm^−1^ and 1630 cm^−1^ signifies the increase in the intensity of C-O stretch mode and suggests that cross linking between functionalized graphene and glutaraldehyde has taken place. The hydroxyl groups can react with aldehyde through formation of hemiacetal structure. The aldehyde functional groups present in the molecule of glutaraldehyde, react with the sheets of functionalized graphene as a cross-linker. Glutaraldehyde binds to the −OH group of the graphite oxide through its one arm containing −CHO group and attaches to the enzyme via lysine amino group through its another −CHO group. Glutaraldehyde is also known to play important role in the self-assembly of graphite oxide nanosheets in aqueous solution, as the self-assembly of nano sheets occur in more ordered fashion in presence of little amount of glutaraldehyde [24]. The FTIR spectra of the enzyme immobilized on functionalized graphene showed peak at 1192 and 1296 cm^−1^, representing carbonyl amide I bond whereas bands at 1481 and 1567 cm^−1^ were observed due to amide II bonds suggesting immobilization of enzyme on functionalized graphene.

### Steady state kinetics

An alteration in kinetic parameters is often brought about by immobilization process due to changes in the microenvironment of immobilized enzyme [25].

**Figure 4:**
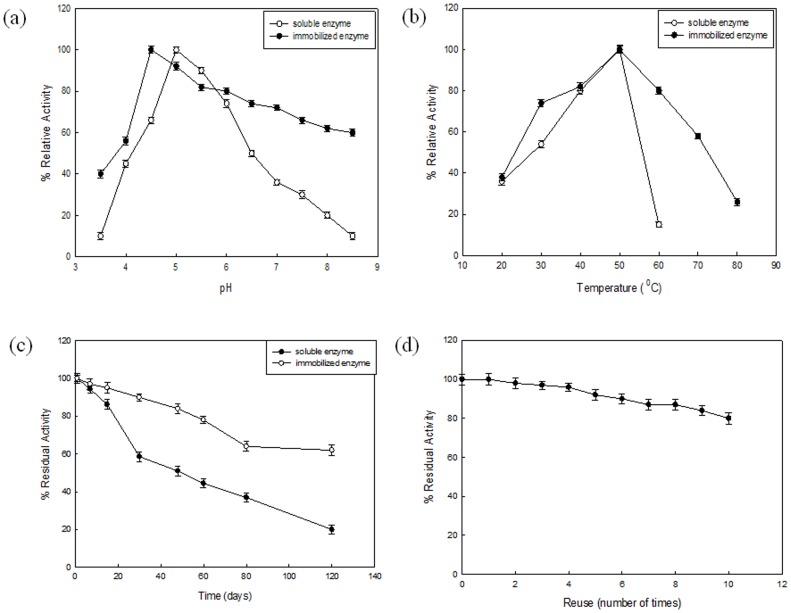
Kinetics, stability and reusability of immobilized enzyme. Effect of pH (a) and temperature (b) on the activity of soluble and immobilized Fenugreek *β*-amylase (c) represents storage stability of soluble and immobilized Fenugreek *β*-amylase and (d) represents reusability (10 uses) of immobilized Fenugreek *β*-amylase.

Fig. 4 (a) shows the comparative pH profile of the soluble and immobilized Fenugreek *β*-amylase with respect to activity and stability. The optimum pH of the immobilized Fenugreek *β*-amylase was found to be 4.5, while the soluble enzyme had pH optima at 5.0. The extent of displacement of optimum pH is influenced by interaction between the enzyme and the matrix and also by the properties of matrix. Hydrolytic activity was observed in a wider pH range by the immobilized enzyme. Compared to soluble enzyme, immobilized one showed better activity in the alkaline range (pH 7.0–9.0). *β*-Amylase from sweet potato immobilized on chitosan and *α*-cyclodextrin also showed similar results [26], [27]. Both immobilized and soluble enzyme showed optimum activity at 50°C (Fig. 4 (b)). Although, immobilized enzyme showed significant enhancement in the tolerance of high temperature. A sharp decline in the activity of soluble enzyme was observed after 50°C, while immobilized enzyme retained 29% activity even at 80°C. Immobilized enzyme showed better thermal stability with retention of around 79% of activity at 65°C when kept for 20 min whereas soluble enzyme lost 86% of enzymatic activity within 5 min when kept at same temperature. Both soluble and immobilized enzyme followed monophasic thermal denaturation kinetics with rate constants 0.2772 min^−1^ and 0.0213 min^−1^, respectively. The immobilization support generally provides protecting effect against enzyme deactivation occurring at high temperature. The immobilization process affects the conformational flexibility and results in increase in enzyme rigidity, which is observed in form of stability towards denaturation resulting from rise in temperature [28]. Enhancement in thermostability of enzyme on being immobilized on nano materials has been shown by others [4], [22]. Unlike the results obtained here, *β*-amylase from soybean showed a small increase in thermostability on being immobilized on chitosan beads [29]. Fenugreek *β*-amylase immobilized on functionalized graphene showed better activity at higher temperatures as compared to *β*-amylase immobilized on phenyl boronate agarose [30] and acrylic carriers [31]. *K_m_* value showed slight change from 1.56 to 1.82 mg/mL on being immobilized on functionalized graphene. These differences can be related to the change in microenvironment resulting from the enzymatic hydrolysis of substrate.

### Storage stability and reusability

Industrial applicability of any enzyme is judged by the knowledge of its storage stability. Stabilization of enzyme by immobilizing it on suitable matrix has been the subject of considerable research, as the intra and inter molecular cross-linking leads to a more rigid molecule that can resist conformational changes. The immobilized enzyme was found to be quite stable when stored at 4°C for 120 days with retention of 60% residual activity, whereas soluble enzyme showed residual activity of 20% during storage for same duration (Fig. 4 (c)). Improved storage stability may result from the reduction in the rate of denaturation of the enzyme on being immobilized.

Another important parameter for industrial application is reusability. Reusability in terms of retention of more than 76% enzymatic activity after 10 repeated uses was shown by Fenugreek *β*-amylase immobilized on functionalized grapheme nanosheets (Fig. 4 (d)). Repeated use can lead to weakening of binding strength between the matrix and the immobilized enzyme resulting in physical loss of enzyme from the matrix. The loss in activity can also be attributed to the distortion of the active site due to recurrent encounter with the substrate causing partial or complete loss of catalytic efficiency.

## Conclusions

Optimization of immobilization using RSM based Box-Behnken design of experiment was proved to be efficient and economical, due to requirement of less number of experiments to be performed. Fenugreek *β*-Amylase covalently attached onto the functionalized graphene showed wider range of pH operability, better thermal stability and greater storage ability as compared to soluble enzyme, making it suitable for production of maltose at industrial scale.
